# Effects of Lengthening Velocity During Eccentric Training on Vastus Lateralis Muscle Hypertrophy

**DOI:** 10.3389/fphys.2019.00957

**Published:** 2019-07-31

**Authors:** Robert Marzilger, Sebastian Bohm, Falk Mersmann, Adamantios Arampatzis

**Affiliations:** ^1^Department of Training and Movement Sciences, Humboldt-Universität zu Berlin, Berlin, Germany; ^2^Berlin School of Movement Science, Humboldt-Universität zu Berlin, Berlin, Germany

**Keywords:** muscle volume, muscle strength, eccentric training, MRI, quadriceps femoris

## Abstract

Eccentric loading is an effective stimulus for muscle hypertrophy and strength gains, however, the effect of lengthening velocity is under debate. The purpose of the current study was to investigate the influence of muscle lengthening velocity during eccentric training on muscle hypertrophy and strength gains at a given overall loading volume. Forty-seven participants were randomly assigned to a control (*n* = 14, age: 26.9 ± 4.1 years) and an experimental group (*n* = 33, age: 27.1 ± 4.4 years). Each leg of the participants in the experimental group was randomly assigned to one of the four eccentric training protocols with different angular velocities (i.e., 45, 120, 210, and 300°/s). Both the magnitude of loading (100% of the isometric maximum) and overall time under tension was matched between the protocols. The training was performed for 33 sessions, 3 times per week with 5 training sets per session. Before and after the intervention, the maximum isometric knee extension moments were measured in all groups using dynamometry, vastus lateralis (VL) muscle anatomical cross-sectional area, and VL muscle volume were measured in the experimental group using magnetic resonance imaging. Data was analyzed in a mixed-design analysis of variance. After the training intervention, the maximum knee joint moments increased in the experimental group (14.2%, *p* < 0.05) but not the control group. VL anatomical cross-sectional area and VL muscle volume increased significantly (*p* < 0.05) in the experimental group (5.1 and 5.7%, respectively), but we did not find any significant differences between the four training protocols in all investigated parameters (*p* > 0.05). The present study provides evidence that muscle hypertrophy and strength gains after eccentric exercise is velocity-independent when load magnitude and overall time under tension are matched between conditions. This is likely due to the similar mechanical demand for the muscle induced by the loading conditions of all four training protocols. The better control of motion and the potentially decreased joint loading compared to high lengthening velocity contractions support the application of slow eccentric exercises in special populations like elderly and people with neurological and musculoskeletal diseases.

## Introduction

Muscle strength is an important contributor to movement performance and quality of live in young, old, and diseased humans (Narici et al., 2003; Moreno Catalá et al., 2013; Mike et al., 2017). Therefore, the maintenance or increase of muscle strength is a basic target in interventions aiming to improve performance during activities of everyday living and particularly, in sports. Improvements in muscle strength are often accompanied by an increase in muscle size, i.e., muscle hypertrophy (Maughan et al., 1983; Kanehisa et al., 1994; O’Brien et al., 2009), because maximum muscle strength is directly related to the physiological cross-sectional area of the muscle (Haxton, 1944). Several training interventions with isometric (Alegre et al., 2014; Noorkõiv et al., 2015), concentric (Blazevich et al., 2007; Moore et al., 2012), eccentric contractions (Paddon-Jones et al., 2001; Shepstone et al., 2005; Moore et al., 2012), or a combination of the three contraction forms (Alegre et al., 2006; Matta et al., 2015) were able to elicit changes in both muscle strength and size (Higbie et al., 1996; Farthing and Chilibeck, 2003; Vikne et al., 2006; Blazevich et al., 2007). When comparing different forms of muscle contractions, eccentric loading was often (Seger et al., 1998; Farthing and Chilibeck, 2003; Vikne et al., 2006; Maeo et al., 2018), but not always (Higbie et al., 1996; Blazevich et al., 2007; Reeves et al., 2009; Cadore et al., 2014), found to be superior in strength improvement and increase in size when compared to training with concentric or isometric muscle loading. This superior effect is often attributed to the higher force that can be generated during eccentric contractions (Westing et al., 1988; Adams et al., 2004) due to the force-velocity relationship (Katz, 1939), resulting in a stronger loading stimulus compared to the other two contraction types. It has been reported that the disruption of muscle sarcomeres (i.e., z-line streaming) is more pronounced after bouts of eccentric contractions when compared to concentric ones (Gibala et al., 1995; Moore et al., 2005). The resultant greater muscle protein syntheses (Moore et al., 2005) and higher satellite cell activation (Crameri et al., 2004) are regarded the most likely mechanisms explaining the greater muscle hypertrophy during eccentric exercise (Franchi et al., 2017).

Earlier studies found that eccentric muscle loading with high lengthening velocity causes more severe muscle damage compared to eccentric contractions with low lengthening velocity (Shepstone et al., 2005; Chapman et al., 2006, 2008). The effect of muscle lengthening velocity after eccentric training on muscle hypertrophy and strength gains was investigated in some studies using maximum voluntary contractions (Paddon-Jones et al., 2001; Farthing and Chilibeck, 2003; Shepstone et al., 2005). However, all these studies used equal number of repetitions between the training conditions leading to a lower time under tension in the training protocol with the fast lengthening contractions. Time under tension is an important component of muscle loading and significantly affects muscle protein synthesis at given load magnitudes (Burd et al., 2012). Therefore, matching the number of repetitions between training protocols, when investigating the effect of lengthening velocity on muscle hypertrophy, may introduce limitations for the interpretation of the outcomes (Chapman et al., 2006). The previous studies (Farthing and Chilibeck, 2003; Shepstone et al., 2005) reported a trend toward greater muscle hypertrophy after eccentric training using fast lengthening contractions, despite a lower time under tension in the fast compared to slow eccentric training. Considering that the time under tension affects muscle protein synthesis (Burd et al., 2012), it can be argued that the non-significant results on the effect of lengthening velocity on muscle hypertrophy (Farthing and Chilibeck, 2003; Shepstone et al., 2005) might have been due to the differences in loading volume between the exercise protocols.

The purpose of the current study was to investigate the influence of muscle lengthening velocity during eccentric exercise on muscle hypertrophy and strength gains by matching the loading magnitude and time under tension in four exercise protocols with different lengthening velocities. We hypothesized a lengthening velocity-dependent muscle hypertrophy and strength improvement after the eccentric training period, with higher gains at faster lengthening contractions.

## Materials and Methods

### Participants

Prior to the intervention, a power analysis according to a similar intervention (Sharifnezhad et al., 2014) indicated that six participants per group were required for a power of 0.95 with an effect size of 0.47 to distinguish group effects. In addition, the dropout rate in the previous intervention was about 40%; hence, we recruited 47 young active (7.4 ± 4 h of sports per week) men to ensure sufficiently sized samples. The participants were acquired from the university population by printed and digital advertising postings, organized primarily by the main experimenter (RM). We were open to include healthy adult males younger than 40 years without any musculoskeletal impairments. The participants were randomly divided into a control (*n* = 14, age: 26.9 ± 4.1 years, body height: 179.0 ± 5.3 cm, body mass: 74.9 ± 7.8 kg) and an experimental group (*n* = 33, age: 27.1 ± 4.4 years, body height: 179.6 ± 6.4 cm, body mass: 73.7 ± 8.8 kg). All participants were informed about the aim and the methods of the study and gave their written informed consent to participate in the investigation. The study was performed in accordance with the declaration of Helsinki and approved by the ethics board of the Humboldt-Universität zu Berlin (EA2/076/15).

### Experimental Design

The participants of the experimental group performed eccentric isokinetic contractions of the knee extensors in a dynamometer (Biodex System 3, Biodex Medical, Inc., Shirley, NY, USA) for 33 trainings sessions, with 3 sessions per week on separate days and 5 training sets per session. The control group did not receive any specific training. The left and right legs of the participants in the experimental group were randomly allocated to one of four training protocols that differed in the movement velocity (protocol p45, angular velocity 45°/s, *n* = 16; p120, angular velocity 120°/s, *n* = 16; p210, angular velocity 210°/s, *n* = 17; p300, angular velocity 300°/s, *n* = 17). The loading magnitude (100% of the isometric maximum voluntary contraction, iMVC) and range of motion (25–100° of knee joint angle; 0° = full extension) were constant between protocols. After each repetition, the leg was returned to the starting position by the participant in the same angular velocity as during loading. Depending on the training velocity, the return of the lever took 1.66 s (p45), 0.63 s (p120), 0.36 s (p210), and 0.25 s (p300), respectively. The training protocols were designed to provide an equal loading volume (i.e. integral of the knee joint moment over the time), and therefore, the number of repetitions was adjusted accordingly, resulting in 3, 8, 14, and 20 repetitions for the protocols p45, p120, p210, and p300, respectively, which provides a similar overall time under tension in all protocols. A 2–3 min break was allowed between the training sets. Training with eccentric contractions is likely to induce muscle soreness (Proske and Morgan, 2001; Chapman et al., 2008). Therefore, we started the training with a rather low loading magnitude of 65% iMVC and gradually increased the load to finally 100% iMVC within the first two training weeks in all experimental protocols. During each training session, the participants were provided with a visual feedback of the target and their generated moment during the eccentric knee extension exercise, to generate the appropriate load magnitude. Due to the higher force potential of a muscle during an eccentric contraction compared to isometric ones, the participants were able to complete all repetitions according to the protocol requirements (i.e., 100% iMVC). The angle-specific target moment was calculated based on a second order polynomial representing the moment-angle relationship. The polynomial was determined in relation to the individual iMVC measurements at 30, 65, and 100° of the knee joint angle. The iMVC measurements were repeated every sixth training session to ensure a progression in the load magnitude.

**Figure 1:**
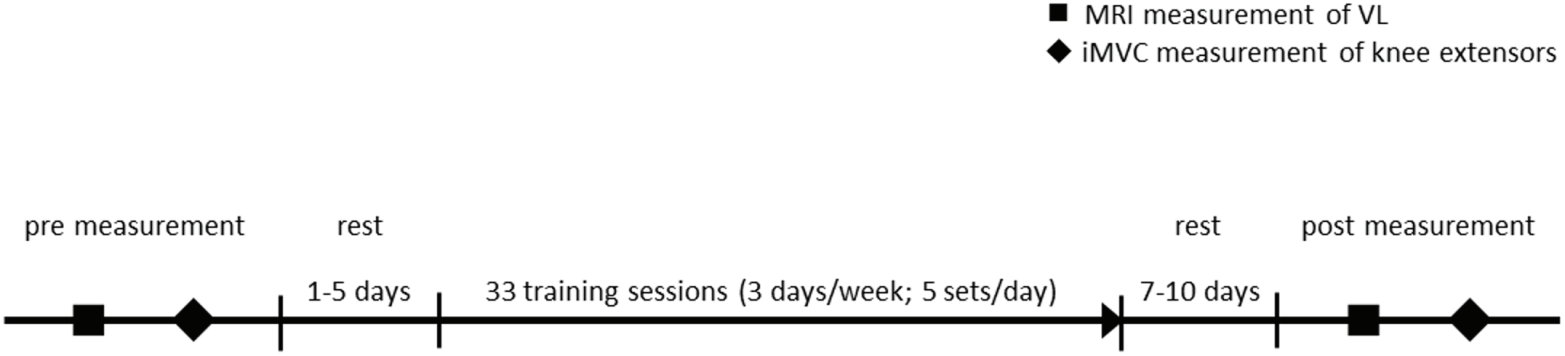
Timeline of the experimental protocol. MRI, magnetic resonance imaging; VL, vastus lateralis; iMVC, isometric maximum voluntary contraction.

The pre and post measurements were done in two separate sessions, 1–3 days apart. In the first session, the vastus lateralis (VL) muscle morphology was measured using magnetic resonance imaging (MRI). During the second session, the maximum knee joint moment of the knee extensors was measured. The MRI-data were collected prior to the measurement of the maximum isometric moment to avoid any bias on the assessment of muscle morphology, e.g., due to muscle swelling from exercise (Clarkson and Hubal, 2002). Further, the post measurements were performed 7–10 days after the last training session, to exclude fatigue effects from the training (Chapman et al., 2006). For cost and time efficiency, the MRI measurements were only performed with the experimental group. Previous studies reported no size-alterations of the quadriceps muscles when training conditions were unchanged (Higbie et al., 1996). A timeline of the measurement is provided in Figure 1.

### Muscle Morphology Measurement

**Figure 2:**
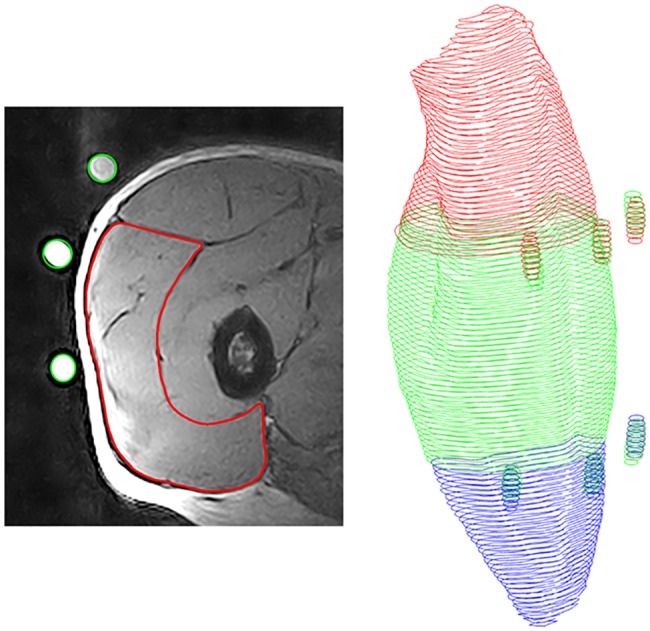
Procedure of muscle reconstruction. Left: Magnetic resonance image of the vastus lateralis with segmented muscle (red) and identified fish oil capsules (green); Right: Reconstructed muscle with fish oil capsules, proximal part (red), medial part (green) and distal part (blue).

The VL muscle was measured and reconstructed using an open 0.25 Tesla MRI scanner (G-Scan, Esaote, Genua, Italy) with the procedure described in detail by Marzilger et al. (2019). Briefly, since the scanner had a small field of view of approximately 20 cm × 20 cm × 20 cm, the thigh was separated with markers (elastic straps with three fish oil capsules) into three sections (distal, medial, and proximal part; Figure 2) based on the femur length and each of these sections was measured separately. The participants were positioned supine in the scanner and three separate Turbo 3D T1 weighted sequences (TE: 16 ms, TR: 39 ms, slice thickness: 3.1 mm, no gaps) from the thigh were acquired. Between the single sequences, the participants were manually repositioned to locate the muscle section of interest into the imaging region of the scanner. To avoid any dislocation of the markers during the repositioning process, the markers were secured with tape and the position was highlighted with a skin marking pen. The segmentation of the MRI images was performed offline using Osirix (version 4.0, Pixmeo, Geneva, Swiss). In a first step, the border (epimysium) of VL was manually segmented in every muscle section (i.e., distal, medial, and proximal). In the second step, the three markers were manually identified in each muscle sequence and an overlap of at least three slices between two sequences (e.g., proximal and medial) was generated. Finally, a custom-written Matlab (2012, The Mathworks, Natick, USA) algorithm calculated the overlap of two different muscle sections due to the position of the identified markers and reconstructed the entire muscle volume from all three muscle sections (Marzilger et al., 2019). From the reconstructed VL muscle, we examined muscle volume, maximum anatomical cross-sectional area (ACSA_Max_) and muscle ACSA in 10% intervals along the muscle length. Due to the small field of view of the MRI scanner, we measured the volume of the VL as representative for the knee extensors due to its great volume and the resultant small relative measurement error in the segmentation process.

### Maximum Knee Joint Moment Measurement

After 10 min of standardized warm-up, including 5 min cycling on an ergometer, as well as several squats and countermovement jumps the participants were seated in the same dynamometer as for the training. The hip angle was set to 85° to reduce the contribution of the bi-articular rectus femoris to the resultant knee joint moment (Herzog et al., 1990). In the following, the participants performed several (in average 4–5) isometric contractions as specific warm-up. The muscle strength was measured during single repetitions of maximal isometric voluntary contractions in five different knee joint angles (60, 65, 70, 75, and 80°). We used these knee joint angles because in this range of motion, the knee extensor muscles generate the maximum joint moment (Mersmann et al., 2014; Sharifnezhad et al., 2014). Due to soft tissue deformation and dynamometer compliance, the real knee joint angles at the plateau of the achieved maximum knee joint moment are different (Arampatzis et al., 2004). Therefore, the measured moments of the dynamometer were corrected for gravitational forces and axis misalignment using the inverse dynamic approach suggested by Arampatzis et al. (2004). For these calculations, the kinematics of each leg was captured with seven cameras (six MX-20 and one F-20) of a vicon motion capture system (Nexus 1.6, Vicon, Stadt, UK). The markers were fixed at the following anatomical landmarks: medial and lateral malleolus, medial and lateral femur condyle, trochanter major and spinae iliaca anterior. Finally, the resultant moments were normalized to body mass and fitted with the corresponding knee joint angles using a second order polynomial to determine the maximal knee joint moment.

### Statistics

All investigated parameters were tested for normal distribution using the Kolmogorov-Smirnov test. A mixed-design analysis of variance for repeated measures (split-plot ANOVA) was performed with time as within-subjects variable (pre vs. post) and exercise protocols as between-subjects factor (p45, p120, p210, p300, and control group). A Bonferroni-Holm corrected *post-hoc* analysis was conducted in the case of a significant interaction of the factors time and protocol. To check the anthropometrical, functional, and morphological parameters of the included participants in each group as well as the percentage pre-to-post changes of muscle morphology and knee joint moment, we used a one-way ANOVA and Bonferroni *post-hoc* comparisons between the groups. The level of significance for all statistical tests was set to *α* = 0.05. In case of significant main effects and interactions, we calculated the partial eta square (*η* ^2^). For respective *post-hoc* observations, we determined Cohen’s *d* effect size for the difference between pre and post values for single protocols.

## Results

Thirteen control participants and 28 participants of the experimental group successfully finished the training and underwent the post measurement, yielding to *n* = 14 participants for each investigated protocol. There was no main effect of group in age (*p* = 0.562), height (*p* = 0.872), and body mass (*p* = 0.860) at the beginning of the intervention. Further, we did not observe any statistically significant changes in body height (*p* = 0.762) and body mass (*p* = 0.157) following the training period.

**Figure 3:**
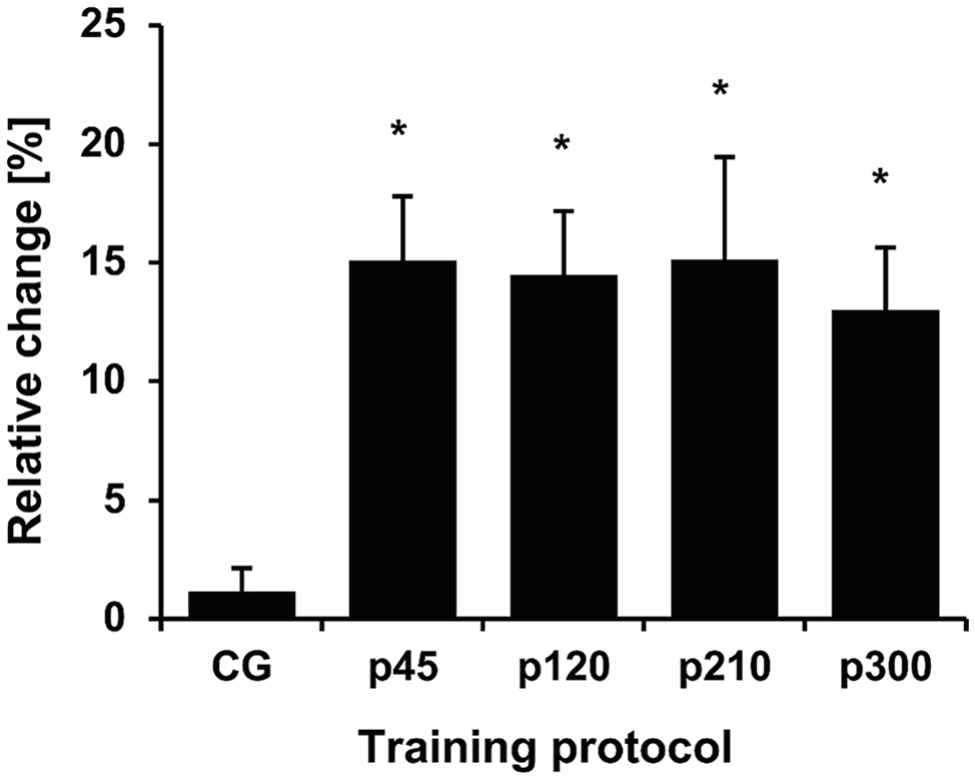
Relative change of the maximum knee joint moment normalized to body mass. The relative changes with regard to baseline are shown on the *y*-axis, while the different protocols are given on the *x*-axis. CG, control group; p45, protocol with 45°/s; p120, protocol with 120°/s; p210, protocol with 210°/s; and p300, protocol with 300°/s. ^*^Statistically significant differences to control group (*p* < 0.05).

**Figure 4:**
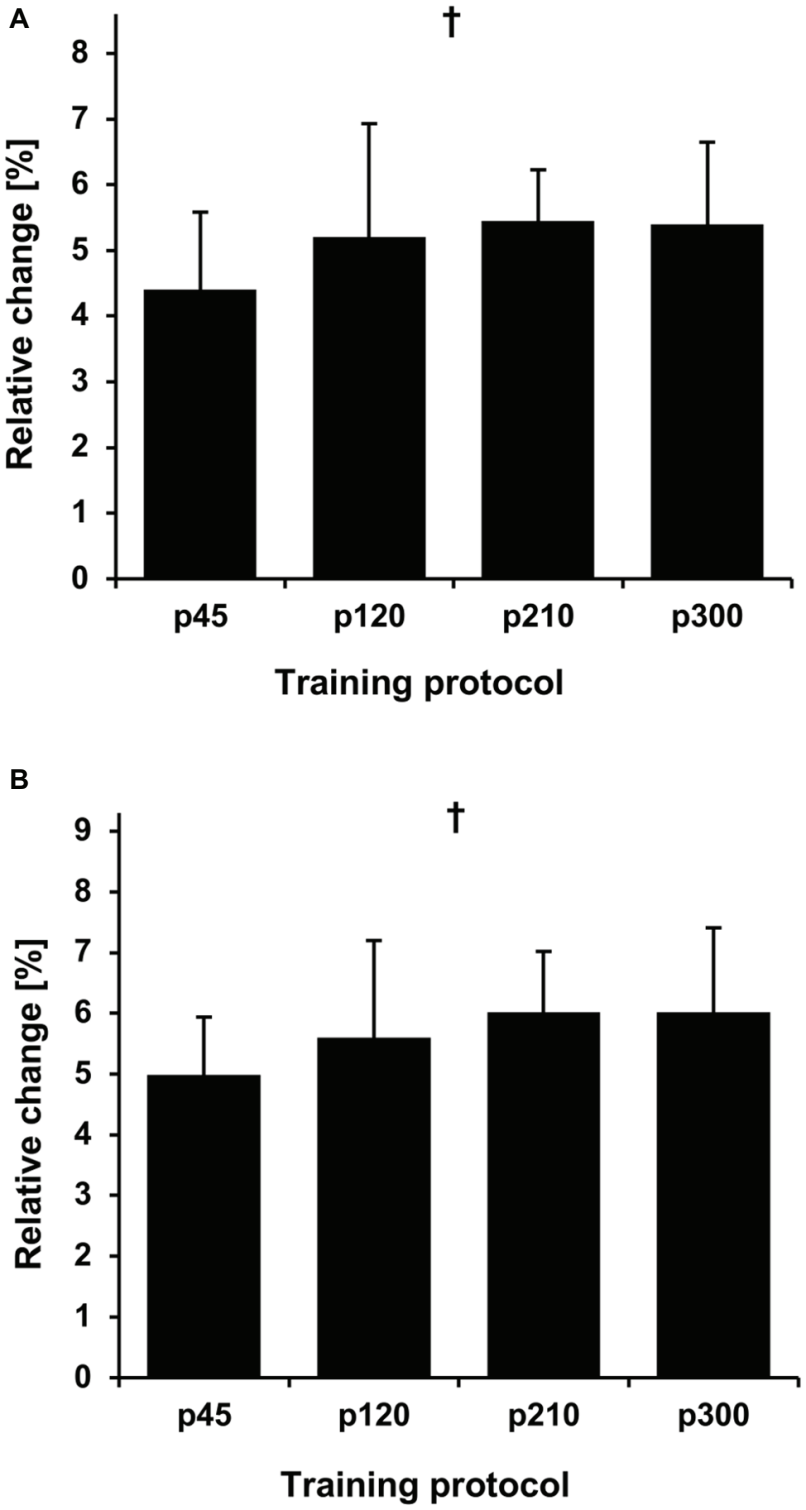
Relative change in maximum anatomical cross-sectional area (ACSA_Max_) **(A)** and muscle volume **(B)** in the four training protocols. The relative changes with regard to baseline are shown on the *y*-axis, while the different protocols are given on the *x*-axis. The error bars indicate the standard error. p45, protocol with 45°/s; p120, protocol with 120°/s; p210, protocol with 210°/s; and p300, protocol with 300°/s. ^†^Statistically significant main effect of time (*p* < 0.001).

**Figure 5:**
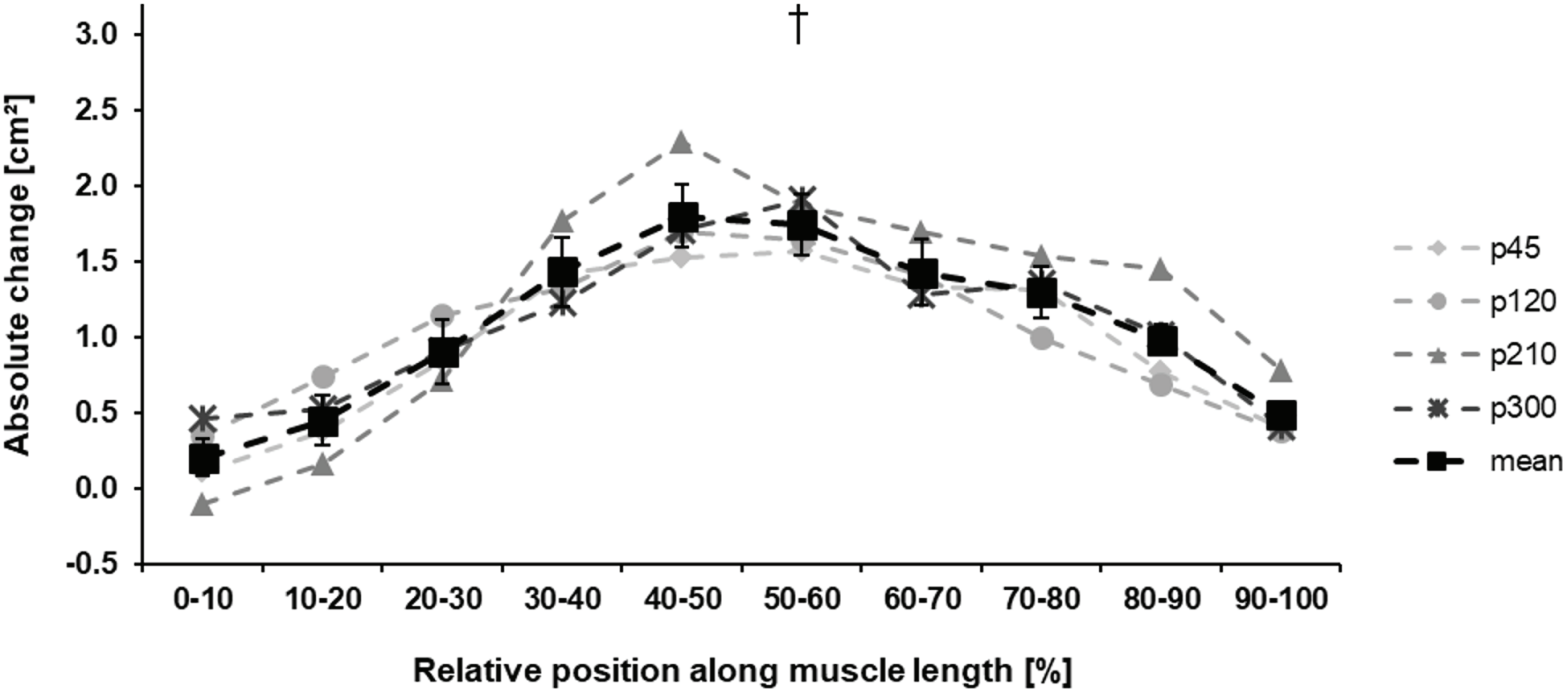
Absolute change ±SE of the anatomical cross-sectional area (ACSA) of the vastus lateralis in the four training protocols along the muscle length. The solid black line (mean) indicates the average change in muscle ACSA of all training protocols (p45, protocol with 45°/s; p120, protocol with 120°/s; p210, protocol with 210°/s; and p300, protocol with 300°/s.). To improve the visibility, the standard error is only shown in the mean data curve. ^†^Statistically significant main effect of time (*p* < 0.001).

The baseline values for all measured parameters are presented in Table 1. Before the training intervention, there were no between-group differences in the maximum knee joint moment (*p* = 0.733), ACSA_Max_ (*p* = 0.570), and muscle volume (*p* = 0.692). The achieved maximum isometric knee extension moment demonstrated a significant effect of time (*p* < 0.001, *η*^2^ = 0.55), with a significant time-by-protocol interaction (*η*^2^ = 0.19, Table 1). The *post-hoc* comparisons showed significant changes in maximum knee joint moment after the training in all experimental protocols but not in the control group (Table 1, Figure 3). The relative changes of maximum moment in the experimental groups (in average 14.2 ± 11.1%) were statistically significant (*p* < 0.05, *post-hoc* comparisons) greater compared to the control group but without any differences (*p* > 0.05) between the training protocols (Figure 3). The ANOVA for both morphological parameters (i.e., ACSA_Max_ and muscle volume) showed a significant effect of time (*p* < 0.001, *η*^2^ = 0.55 for ACSA_Max_ and *η*^2^ = 0.65 for muscle volume), but no time-by-protocol interaction, indicating no differences in the training-induced alterations between the four training protocols. The average increases in ACSA_Max_ and muscle volume were 5.1 ± 4.7% and 5.7 ± 4.6%, respectively (Figure 4). In a similar manner, there was a significant increase (*p* < 0.001 to *p* = 0.008) of the ACSA in every 10% interval (except from 0 to 10%) along the VL muscle length without any time-by-protocol interactions (*p* > 0.05; Figure 5).

**Table 1:** Mean ± SD of the maximum knee joint moment (Moment_Max_), maximum anatomical cross-sectional area (ACSA_Max_) and muscle volume for the control (CG) and experimental groups (p45, protocol with 45°/s; p120, protocol with 120°/s; p210, protocol with 210°/s; p300, protocol with 300°/s); *d*, Cohen’s *d*; Range, range of change.

		Control group	Experimental group			
		CG	p45	p120	p210	p300
Moment_Max_ (Nm/kg)^†^ ^,^ ^#^	Pre	3.7 ± 0.5	3.7 ± 0.4	3.9 ± 0.4	3.9 ± 0.4	3.9 ± 0.4
	Post	3.7 ± 0.4	4.3 ± 0.5^*^	4.4 ± 0.4^*^	4.4 ± 0.7^*^	4.4 ± 0.5^*^
	Range	−0.3–0.3	−0.2–1.1	−0.1–1.1	−0.4–2.0	−0.2–1.1
	*d*		1.213	1.293	0.921	1.072
ACSA_Max_ (cm^2^)^†^	Pre	–	35.9 ± 2.7	34.4 ± 4.9	34.4 ± 5.4	33.5 ± 4.8
	Post	–	37.5 ± 3.2	36.1 ± 4.8	36.4 ± 6.0	35.3 ± 5.5
	Range		−1.3–4.4	−2.3–5.5	0.1–3.7	−1.3–5.6
	*d*		0.505	0.347	0.273	0.320
Muscle volume (cm^3^)^†^	Pre	–	744.0 ± 82.5	715.4 ± 113.6	742.4 ± 143.9	699.1 ± 118.1
	Post	–	780.9 ± 89.6	752.9 ± 108.1	784.8 ± 143.7	739.9 ± 121.0
	Range		−6.4–81.3	−20.2–112.5	−4.8–71.8	−43.7–85.9
	*d*		0.416	0.334	0.297	0.342

† *Statistically significant main effect of time (p < 0.05)*.

# *Statistically significant time-by-protocol interaction (p < 0.05)*.

* *Statistically significant differences (post-hoc analysis) between pre und post measurement (p < 0.05)*.

## Discussion

The present study shows that an exercise intervention using eccentric contractions with a high load magnitude (100% of iMVC) and a relative large range of motion (75°) provides an effective stimulus for VL muscle hypertrophy of up to 5% in 11 training weeks and can improve knee extensors muscle strength by up to 14%. Since no differences between the training protocols were observed, the results are in contrast to our hypothesis and provide evidence that muscle hypertrophy is independent of the contraction velocity applied during eccentric exercise. Therefore, our hypothesis of a lengthening velocity-dependent muscle hypertrophy needs to be rejected.

Several studies found an increase in maximum muscle strength (Farthing and Chilibeck, 2003; Vikne et al., 2006; Blazevich et al., 2007; Moore et al., 2012), anatomical cross-sectional area (Higbie et al., 1996; Vikne et al., 2006; Moore et al., 2012), and muscle volume (Blazevich et al., 2007) after training with eccentric contractions. Some earlier studies investigating the effects of lengthening velocity on muscle adaptation after a period of training concluded that eccentric exercise using fast lengthening contractions (i.e., 180 or 210°/s) lead to greater muscle hypertrophy and strength gains compared to slow (i.e., 20 or 30°/s) contractions (Paddon-Jones et al., 2001; Farthing and Chilibeck, 2003; Shepstone et al., 2005). The greater muscle damage that usually occurs during the fast lengthening contractions and the resultant greater muscle remodeling and greater duration of protein synthesis (Farthing and Chilibeck, 2003; Shepstone et al., 2005) was suggested as a possible mechanism for this phenomenon (i.e., fast eccentric contractions being more effective for muscle hypertrophy and strength gains). A greater muscle hypertrophy during the fast lengthening contractions (Farthing and Chilibeck, 2003; Shepstone et al., 2005) was, however, not statistically confirmed (both studies found only a trend toward greater muscle hypertrophy). Furthermore, in these earlier studies, the time under tension during the training was different between protocols, and therefore, it is not possible to isolate the effects of the lengthening velocity on muscle hypertrophy. Unequal times under tension in muscle loading result in a different stimulation of muscle protein synthesis, which in turn affects muscle hypertrophy after exercise (Burd et al., 2012).

In our experiment, we applied the same level of load magnitude and total time under tension on the knee extensor muscles and we used an equal range of motion during the eccentric contractions. The only difference between the four investigated exercise protocols was the lengthening velocity. The angular velocities ranged from 45 to 300°/s and represent a wide range of lengthening velocities. As there is a good reason to assume that training volume shows a direct influence on training outcomes (Mitchell et al., 2012), we can argue that the used experimental design is most suitable to investigate the isolated effects of lengthening velocity on muscle hypertrophy and muscle strength gains after eccentric training and, to our knowledge, this is the first longitudinal study applying this approach. Additional to the similar increase in muscle volume and muscle strength in all four protocols, we did not find any specific regional muscle hypertrophy in any of the different protocols as reported in other studies investigating hypertrophic muscle responses by comparing eccentric and concentric training (Blazevich et al., 2007; Franchi et al., 2014). Although the absolute ACSA in every 10% interval along the muscle length increased, we did not find any differences between the protocols.

Our findings provide evidence for an independence of the exercise-induced muscle hypertrophy and muscle strength from lengthening velocity and support the idea that magnitude of loading (Sharifnezhad et al., 2014), muscle length at which the load is applied (Sharifnezhad et al., 2014; Noorkõiv et al., 2015) and time under tension might be the main determinants of muscle adaptation after eccentric training. We hypothesized that muscle hypertrophy would be preferentially increased in the training protocols with the high lengthening velocities based on the reports of greater muscle damage in fast eccentric contractions (Shepstone et al., 2005; Chapman et al., 2006) and the resultant greater muscle remodeling (Farthing and Chilibeck, 2003; Shepstone et al., 2005). However, the damage-based muscle hypertrophy is currently in debate and there is experimental evidence of muscle hypertrophy without previous muscle damage (Schoenfeld, 2012; Hyldahl and Hubal, 2014; Franchi et al., 2017). It has been reported that the titin kinase domain can be directly activated by mechanical force to trigger signaling independent of muscle damage after eccentric loading (Lange et al., 2006; Puchner et al., 2008). Furthermore, it is well known that muscles adapt to the exercise-induced damage after eccentric loading, the so called repeated bout effect, which provides protection against further muscle damage (Nosaka and Clarkson, 1995; Hyldahl et al., 2015). Neural adaptations in the motor unit recruitment, extracellular matrix alterations initiated by the activation of specific extracellular matrix signaling pathways, changes in the fascicle kinetics and increased inflammation sensitivity are important mediators to the protective effects against further muscle damage (Hyldahl et al., 2015, 2017). In our study, the participants successfully completed 33 training units over 11 weeks of eccentric training at 100% of the iMVC. Although we did not investigate muscle damage in our experiment, we can assume that, due to the repeated bout effect and the protective muscle adaptation against eccentric loading-induced damage the observed muscle hypertrophy was not mainly due to muscle damage. Recent studies (Franchi et al., 2014, 2015) reported similar muscle hypertrophy gains and similar long-term muscle protein synthesis after eccentric and concentric training when the relative load was matched between the two different contraction modes. We argue that the matched loading magnitude, time under tension, and range of motion used in our protocols were the reasons for the similar and velocity-independent hypertrophic responses in the current investigation.

The increase in muscle strength can be attributed to both the observed muscle hypertrophy and neural adaptations (Higbie et al., 1996; Paddon-Jones et al., 2001; Douglas et al., 2017). The greater effects observed on strength compared to the morphological changes suggest a considerable neural contribution. However, as both muscle hypertrophy and strength development were similar between protocols, we also would expect a similar contribution of neural adaptation, which is likely explained by the same intensity (in terms of force production) across protocols (Campos et al., 2002). Though in our study no differences in the hypertrophic response between protocols were observed, it might still be that structural changes within the muscle occurred dependent on the lengthening velocity during exercise. It has been reported that after acute eccentric loading mostly the fast glycolytic fiber type demonstrated histologic abnormalities (Fridén et al., 1983; Lieber and Fridén, 1988), resulting in selective increased satellite cell activation (Cermak et al., 2013; Hyldahl et al., 2014) and hypertrophy of type II fibers (Hortobágyi et al., 2000; Verdijk et al., 2009). The selective fiber type II specific hypertrophy after long term eccentric exercise is pronounced at fast lengthening contractions (Paddon-Jones et al., 2001; Shepstone et al., 2005). However, to what extent fiber type-specific hypertrophy contributed to the overall similar increases in muscle volume of the four training protocols cannot be answered with our present study design.

Due to the present study design, a possible cross-education effect between limbs cannot be ruled out completely. However, there is no scientific evidence for interlimb transfer effects in terms of exercise-induced muscle hypertrophy (Lee and Carroll, 2007; Hendy and Lamon, 2017). Thus, any possible transfer effect is limited to strength gains as a result of neural adaptation. Yet, neural adaptation shows considerable specificity to the type and velocity of a muscle contraction (Hortobágyi et al., 1996; Dos Santos Rocha et al., 2011), which suggests a reduced contribution of cross-education as a result of eccentric training on isometric strength as measured in our study. Moreover, cross-education shows strong effects on untrained contra-lateral limbs, yet if the contra-lateral limb also receives training, it seems likely that the neural activity associated to it largely eliminates the cross-education effect. Moreover, the combination of the exercise protocols within the current intervention was completely randomized between participants, and thus, any combination of protocols for the two legs was possible (e.g., 45 and 300°/s or 45 and 210°/s or 210 and 120°/s, …). Therefore, any possible cross-transfer effects would also be randomly distributed over the applied protocols and not systematically bias our results.

In conclusion, the current study demonstrated that muscle hypertrophy after eccentric training interventions is velocity-independent when time under tension is matched between conditions and the load magnitude and range of motion are similar. Gains in muscle volume directly affect the maximum muscle power (Narici, 1999; O’Brien et al., 2009), which is of major importance for both sports performance in disciplines involving jumping and sprinting (Cronin and Sleivert, 2005) as well as for mobility functions in the elderly (Tschopp et al., 2011; Izquierdo and Cadore, 2014). However, lower movement velocities allow for a better control of motion and lower fluctuations of accelerations during eccentric contractions (Christou et al., 2003), which might reduce peak forces in the involved joints given that controlled movement behavior plays a major role for joint loads (Bergmann et al., 2004). Further, the longer time under tension during single repetitions in slow eccentric training might provide a superior stimulus for tendon adaptation compared to fast eccentric movements (Arampatzis et al., 2010; Bohm et al., 2014), which might be important for the prevention of imbalances of muscle and tendon adaptation (Mersmann et al., 2017, for a review). Therefore, we argue that the application of eccentric exercises with low angular velocities could be more applicable for muscle training in populations like elderly, people with neurological and musculoskeletal diseases or athletes at risk for tendinopathy (Hyldahl and Hubal, 2014; Mersmann et al., 2017).

## Data Availability

The datasets generated for this study are available on request to the corresponding author.

## Ethics Statement

All participants were informed about the aim and the methods of the study and gave their written informed consent to participate in the investigation. The study was performed in accordance with the declaration of Helsinki and approved by the ethics board of the Humboldt-Universität zu Berlin (EA2/076/15).

## Author Contributions

RM and AA conceived the experiment. RM and SB performed the experiments. RM analyzed the data and SB, FM, and AA substantially contributed to data analysis. RM, SB, and AA interpreted the data. RM and AA drafted the manuscript, and SB and FM made important intellectual contributions during revision. All authors approved the final version of the manuscript and agree to be accountable for the content of the work.

### Conflict of Interest Statement

The authors declare that the research was conducted in the absence of any commercial or financial relationships that could be construed as a potential conflict of interest.
